# Towards Design Automation of Microfluidic Mixers: Leveraging Reinforcement Learning and Artificial Neural Networks

**DOI:** 10.3390/mi15070901

**Published:** 2024-07-10

**Authors:** Yuwei Chen, Taotao Sun, Zhenya Liu, Yidan Zhang, Junchao Wang

**Affiliations:** School of Integrated Circuit Science and Engineering, Hangzhou Dianzi University, Hangzhou 310018, China

**Keywords:** microfluidic mixers, reinforcement learning, design automation

## Abstract

Microfluidic mixers, a pivotal application of microfluidic technology, are primarily utilized for the rapid amalgamation of diverse samples within microscale devices. Given the intricacy of their design processes and the substantial expertise required from designers, the intelligent automation of microfluidic mixer design has garnered significant attention. This paper discusses an approach that integrates artificial neural networks (ANNs) with reinforcement learning techniques to automate the dimensional parameter design of microfluidic mixers. In this study, we selected two typical microfluidic mixer structures for testing and trained two neural network models, both highly precise and cost-efficient, as alternatives to traditional, time-consuming finite-element simulations using up to 10,000 sets of COMSOL simulation data. By defining effective state evaluation functions for the reinforcement learning agents, we utilized the trained agents to successfully validate the automated design of dimensional parameters for these mixer structures. The tests demonstrated that the first mixer model could be automatically optimized in just 0.129 s, and the second in 0.169 s, significantly reducing the time compared to manual design. The simulation results validated the potential of reinforcement learning techniques in the automated design of microfluidic mixers, offering a new solution in this field.

## 1. Introduction

Microfluidics is a technology that precisely controls fluids at the micrometer scale [1], capable of handling objects ranging from several hundred micrometers [2] to a few micrometers in size. This technology is extensively applied in various fields including bioengineering [3], chemical experimentation [4], and medical diagnostics [5]. Microfluidic chips are typically made from glass, silicon, or polymers and feature finely fabricated microchannels that automate sample preparation [6,7], reactions, separations [8,9], and detections [10,11]. The advantages of this technology include low cost, high precision, and high throughput, making it suitable for applications such as droplet generation [12,13], cell sorting [14,15,16], drug screening [17,18,19], organs-on-a-chip [20,21,22], single-cell RNA sequencing [23,24], and real-time diagnostics [25].

Micromixers represent an application of microfluidic technology, primarily utilized for the rapid mixing of different samples within microscale devices [26,27,28]. Micromixers are categorized into active and passive types. Active micromixers enhance mixing efficiency [29] through external energy sources such as magnetism [30], acoustics [31], thermal energy [32], or electricity [33]; however, this may increase system complexity and cost. In contrast, passive micromixers increase fluid contact area and time by designing special microchannels, which may lower mixing efficiency, but offer advantages in terms of lower cost, reduced complexity, and ease of manufacture [34]. When designing micromixers, precise control over the dimensions and shapes of the micromixers is essential to ensure accuracy in experimental outcomes and mixing efficiency, utilizing the layout or the geometry of the microchannel structure in the chip to generate a specific fluid profile, which is helpful for the mixing phenomena [34].

Moreover, under conventional application scenarios, the traditional methods of designing and applying micromixers are not only time-consuming and labor-intensive, but also, the design work is typically restricted to experts in the field [35]. Concurrently, the evolution of micromixers to meet emerging demands continues to be an iterative process characterized by “trial and error”. This necessitates that researchers dedicate substantial time to numerous repetitive design simulations to ascertain a reliable and stable micromixer chip architecture that aligns with the required dimensional specifications. Such a method can be both time-intensive and financially burdensome, particularly in complex or extreme scenarios. This inefficient chip design method not only limits the potential application scenarios of current micromixers, but also significantly impacts their prospects in clinical testing and industrial production. Consequently, many developers are urgently seeking to implement an automated microfluidic chip design process, hoping that this automated scheme will expand the utility and application range of microfluidic mixers. Therefore, to truly leverage the existing capabilities of microfluidic technology and promote its broader application in the future, it is imperative to explore a viable automated design solution for microfluidic mixers.

Thanks to the vigorous development of artificial intelligence (AI) technology in recent years, increasingly sophisticated AI techniques have provided researchers with new avenues for automating the design of micromixer chips [36]. Granados-Ortiz defines a Machine Learning-Assisted Design Optimization (MLADO) approach that deals with a multi-objective optimization problem by means of a Random Forest classifier, using genetic algorithms to optimize the agent model, thus obtaining the optimal geometrical configuration of the mixer [37]. AI technology can utilize trainable statistical models for pattern recognition and future behavior prediction, marking a novel direction for research into automated microfluidic mixer design [38]. D. de Oliveira Maionchi employed a neural network to simulate and analyze how the diameter and offset of obstacles influence mixing efficiency, pressure drop, and energy consumption. By training the dataset with this method, the research conclusively identified the optimal size and positioning of obstacles using a genetic algorithm [39].

Currently, the field of AI-based automation in microfluidic design has garnered considerable attention. Our previous work [40] transformed fluid dynamics problems into image-recognition challenges, employing a convolutional neural network (CNN)-based technique to predict the fluid behavior of stochastic microfluidic mixers. This approach facilitated micromixer chip design through a reverse engineering process, where optimization algorithms iteratively refine the model to determine the optimal design. Researchers like MT Birtek [41] have optimized the design of non-Newtonian microfluidic systems by using algorithms that process input from a training library and generate predictions before manufacturing, significantly reducing development costs and time. Granados-Ortiz [37] and colleagues designed an efficient mixing mechanism featuring a rectangular pillar placed within a microchannel, with simulation data showing that this design surpasses the efficiency of previously reported devices. Lashkaripour [42] introduced a neural network-based fluid dynamics automation tool (DAFD), aimed at predicting performance and automating the design of focused flow droplet generators. This tool demonstrated significant capabilities in achieving user-specified performance metrics, closely matching the desired diameter and rate with errors of 4.2% and 11.5%, respectively. It also showed potential for adapting to different fluid combinations without the need for extensive datasets. Khor [43] and his team utilized machine learning to discover a low-dimensional code encapsulating droplet shapes, further predicting the likelihood of droplet rupture. Their method achieved an impressive 91.7% classification accuracy in predicting droplet rupture, a significant improvement from the traditional accuracy of about 60%.

Currently, there exists no universally acknowledged methodology for the design of automation solutions for micromixers that garners consensus among all, or even the vast majority, of practitioners. This indicates that the existing automation algorithms have yet to be refined to satisfy all market demands, thereby highlighting the ongoing potential for research and advancement in this domain. In other words, there is no doubt that a more intelligent and universal automatic design algorithm is needed.

Currently, there is no industry consensus on a rapid, efficient, and standardized design method for micromixers. This lack of a standardized approach means that researchers designing a new micromixer often have to invest significant human and material resources if they cannot match an efficient design scheme, and the final design results may not even meet the basic expected requirements. Therefore, this paper aims to develop a new computer algorithm process capable of automatically designing the dimensional parameters of micromixers, providing researchers with more guidance in selecting design options when designing micromixer chips.

In recent years, reinforcement learning has been extensively applied in fields such as chess games [44], flight control [45], robotic control [46], game strategies [47], and urban traffic management [48]. Therefore, the mechanization design of a micromixer using reinforcement learning technology is a viable avenue for research. Simultaneously, owing to the distinctive technical advantages of reinforcement learning technology, the mechanization approach of micromixers based on reinforcement learning technology can be employed to address decision-making challenges in intricate environments and engage in autonomous learning and gradual optimization through interaction with the environment. Furthermore, this type of automated design algorithm possesses the capability to generalize in the design process, thereby enabling more flexible application across a variety of design scenarios.

In this study, we propose an automatic design algorithm for micromixer chip sizing based on reinforcement learning techniques. The intelligent agents trained by this algorithm can quickly analyze state sets in different environments, make action decisions based on reward function calculations, and efficiently design micromixer chip dimensions. COMSOL is an advanced simulation tool for computational fluid dynamics issues, utilized to simulate and evaluate the mixing effects of various micromixer designs. Given the necessity to conduct numerous COMSOL simulations to gather data during the reinforcement learning process, frequent invocations of these simulations prove to be excessively time-consuming, rendering the reinforcement learning algorithms for state evaluation computationally burdensome and inefficient. To address this, we collected data from a limited set of COMSOL simulations of the test structures and trained an artificial neural network with these data. These data were employed to develop a low-overhead neural network model that predicts different structural parameters and simulates the mixing efficiency of micromixer chips under various conditions. Ultimately, this algorithm was applied to successfully automate the design of two basic micromixer chip structures, achieving satisfactory design outcomes.

The micromixer chip design process introduced in this paper, leveraging reinforcement learning, significantly reduces the human and material resources needed to achieve designs that meet the expected standards. Moreover, the application of this algorithm also lowers the overall design complexity and the skill requirements for designers. This enhances the broader applicability and potential value of this design approach to the automated design of micromixers.

## 2. Methods of the Design

### 2.1. Design and Theory

**Figure 1:**
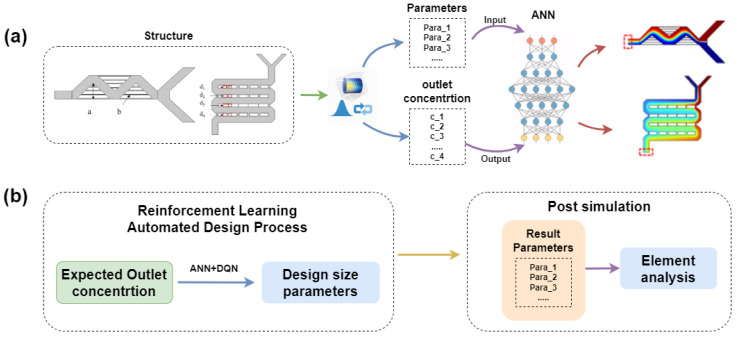
The flowchart of the automated design of micromixer using reinforcement learning technology (**a**) used to train an artificial neural network for predicting the outlet concentration of the mixer. (**b**) A flowchart for designing optimal size parameters and post-simulation verification using reinforcement learning technology.

This study introduces an automated design process for microfluidic mixer size parameters for different expected mixing concentrations based on a reinforcement learning algorithm, as shown in Figure 1. To minimize the impact of different size parameters on the structure’s mixing concentration outcomes, we employed an artificial neural network (ANN) to predict the corresponding simulation results. Figure 1a presents the flowchart of the ANN model used to predict the mixing concentration results for different size parameters. The design dimensions of the micromixer serve as the input parameters to the neural network model, while the concentration simulation results at the corresponding output ports are used as the output parameters of the model. Through training, we have developed a high-precision, low-overhead ANN model capable of predicting outlet concentrations based on mixer design parameters. Within the entire automated design algorithm, optimal design parameters corresponding to specified target outlet concentration values can be obtained using a reinforcement learning agent. Subsequently, using reinforcement learning techniques and our defined evaluation function, the algorithm automatically optimizes the size parameters for different performance metrics, followed by post-simulation to verify the performance of the reinforcement learning automated design algorithm, as illustrated in Figure 1b.

### 2.2. Experimental Subjects

**Figure 2:**
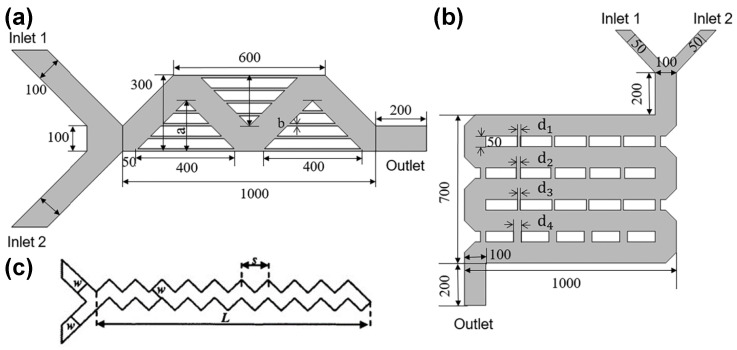
The geometric structures of two mixer designs: (**a**) the structure of the mixer design 1, (**b**) the structure of the mixer design 2, and (**c**) the Y-mixer geometry.

This study selected two classic micromixers as examples and used COMSOL Multiphysics to simulate the concentration fields of the micromixers. As shown in Figure 2a, this is a reference view of the mixer design 1 geometry from the top. The structure has two inlet channels, each 100 microns wide, an outlet 100 microns wide, and three triangular mixing zones with four microchannels, each 5 microns wide, evenly distributed within each triangle. The specific parameters within this mixing zone can be found in Figure 2a. The values of a and b are variable. The design incorporates sawtooth microchannels of uniform width, set at a 90-degree angle to each other. a denotes the linear length of the periodic step, and L represents the linear length of the Z-shaped microchannel. This is the classic Y-sawtooth microchannel structure, a typical passive micromixer structural design [49]. The parameter s is the linear length of the periodic step, and the parameter w is the linear width of the channel, as shown in Figure 2c. To simplify the analysis, the dimensionless parameter s/w is adjusted to enhance the mixing efficiency (the ratio of the minimum to maximum concentrations at the outlet cross-section, $C_{min}$/$C_{max}$).

The geometric shape of mixer design 2 is illustrated in Figure 2b, featuring two inlets each 50 micrometers wide, a 100-micrometer-wide outlet, and a rectangular mixing area measuring 700 × 1000 micrometers. Within this area, a 4 × 5 rectangular array with a height of 50 micrometers is created, with each row of microchannels (the spacing between rectangles) being uniformly wide. Four parameters, $d_{1}$, $d_{2}$, $d_{3}$, and $d_{4}$, are randomly set within a specified range.

The primary objective of this research is to test the feasibility of the automated design algorithm proposed in this paper, rather than to design the mixers themselves. Therefore, for practical testing, two variables were selected for design 1 and four variables for design 2, instead of treating all other parameters as variable, to conduct a proof-of-concept study. Additionally, the chosen parameters capture the main geometric features of the mixing zone, which are highly relevant to the performance of the proposed mixer designs. The range of variable parameters for both structures is shown in Table 1.

### 2.3. Low-Cost Model Training for Micromixers Based on Artificial Neural Networks

In this study, artificial neural networks trained through supervised learning will be used instead of finite-element analysis to predict the concentrations at the outlets of the micromixers. Using artificial neural networks to predict outlet concentrations will significantly enhance the analytical efficiency of micromixer designs and optimize the training processes for reinforcement learning agents.

#### 2.3.1. Neural Network Training Data Collection

Based on the variable structural dimensions of the two classic mixer geometries introduced in Section 2.2, mixer design 1 was configured with two variable parameters that vary the width of the main channel of mixer design 1. In mixer design 2, a rectangular array of four rows and five columns was constructed. The value ranges of the variables are shown in Table 1.

By importing the geometric structures of the two mixer designs into COMSOL, and then using the laminar flow physics module and the dilute species transport physics module to simulate the velocity and concentration fields, respectively, two fixed solvers were set up to solve the concentration distribution of the two mixer designs. The variable parameters of each mixer design and the concentration values at 11 outlet points were converted into matrices, with the mixer design parameters varied within a certain range (refer to Table 1). Each mixer design explored a sufficient number (sampling 10,000 designs each) of training designs for later stages, and the corresponding datasets and simulation models were stored in a local database for further use.

**Figure 3:**
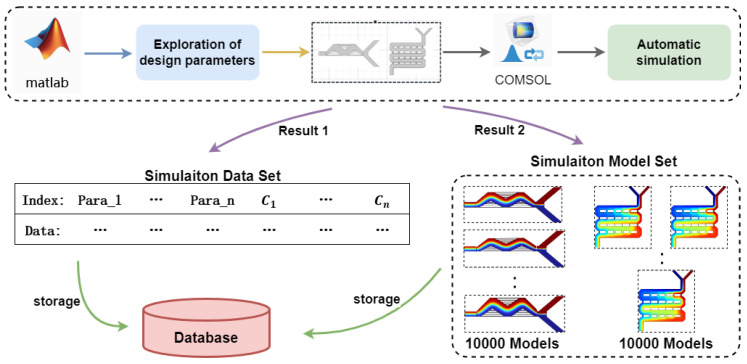
The database construction process of classic mixers design.

To facilitate the use of artificial neural networks to predict the outlet concentration of mixer designs, this section utilizes finite-element simulation software to build a database, the data from which will be used to train the neural network models. The specific process is illustrated in Figure 3.

#### 2.3.2. Data Preprocessing and Neural Network Training

Data preprocessing constitutes a vital phase in the training of artificial neural networks. To bolster the predictive accuracy of our neural network model, we opted for max–min normalization as our mode of data preprocessing in this study. The precise formula is presented as Equation (1):

(1) $$x^{\prime }=\frac{x-x_{min}}{x_{max}-x_{min}}$$

In Equation (1), $x_{min}$ represents the minimum value of the feature data within the column, $x_{max}$ is the maximum value, and $x^{\prime }$ is the raw data to be processed. After normalization, the data are transformed to fall within the range of [0, 1].

### 2.4. Using Reinforcement Learning Techniques for Micromixer Structural Dimension Design

#### 2.4.1. Performance Evaluation Methods for Model Predictions of Outlet Concentration across Design States

A direct analysis is necessary to determine whether different design parameters are excellent or poor, and among those deemed excellent, which specific design is superior. To assess the quality of different design parameters, it is necessary to define an evaluation function that assigns scores to different design options based on their dimensions, reflecting the merits and demerits of different practical schemes.

To quantitatively describe the mixing efficiency of the micromixers, the mixing index is utilized, which measures the standard deviation of molar concentration across a cross-section [50].

It is worth noting that the mixing effect of the micromixer is affected by the Reynolds number and Péclet number in practical applications. In this experiment, we set the single-port inlet flow rate to 10 µL/min, which is usually the standard flow rate for operation in different scenarios according to different purposes [51]. Also, we set the boundary condition of the outlet to a pressure of 0 Pa. In addition, the solute diffusion coefficient of fluorescein (4.25 × $10^{-10}$ $m^{2}$$s^{-1}$) was used in the experiment, and the density and viscosity of the fluid were set to parameters characteristic of water at room temperature: the fluid density was set to 1000 kg/$m^{3}$, and the dynamic viscosity of the fluid was set to 1 mPa·s.

(2) Re=ρuLμ

(3) $$\mathrm{Pe}=\frac{uL}{D}$$

In Equations (2) [52] and (3) [53], ρ denotes the density of the fluid, *u* denotes the characteristic velocity, *L* denotes the characteristic length, μ denotes the dynamic viscosity of the fluid, and *D* denotes the diffusion coefficient.

We can calculate the Reynolds flow under the simulated conditions so that we can estimate the Re number and Pe number corresponding to the conditions of this experiment. According to Equation (2), the Reynolds number (Re) is 0.001, while according to Equation (3), the Péclet number (Pe) is approximately 2.35.

Nonetheless, the primary focus of this study is to explore the potential of reinforcement learning techniques in optimizing the dimensions of micromixers. For this research, the fluid flow characteristics within the mixer are consistently controlled during the experimental procedures. The calculation is represented by the following formula:

(4) $$\delta =\frac{\sqrt{\frac{1}{N}\sum_{i=1}^{N}{\left(c_{i}-\bar{c}\right)}^{2}}}{\sqrt{\frac{1}{N}\sum_{i=1}^{N}{\left(c_{0}-\bar{c}\right)}^{2}}}$$

In Equation (4), *N* represents the total number of sampling points on the cross-section, $c_{i}$ is the molar concentration at sampling point *i*, and $\bar{c}$ is the average molar concentration when the sample is completely mixed, usually set at 0.5. $c_{0}$ indicates the concentration in the case of no mixing, typically valued at 0 or 1. σ represents the deviation of molar concentrations at sampling points from the fully mixed average.

(5) ϵ=1−σ

In Equation (5), the mixing index, a number between 0 and 1, is introduced. When it is close to 1, it indicates that the fluids in the mixer are fully mixed; when it is close to 0, it indicates that the fluids in the mixer are not mixed; thus, it is used to represent the mixing performance of the mixer.

#### 2.4.2. Theoretical Foundations of Automated Design Combined with Reinforcement Learning Algorithms

Reinforcement learning, inspired by behavioral psychology [54], mimics biological learning patterns by memorizing scenarios through behaviors reinforced by rewards, making it easier to repeat these behaviors upon re-encounter. Unlike supervised and unsupervised learning, which depend on pre-existing data, reinforcement learning generates data through interactions with the environment to seek optimal strategies.

In the process of reinforcement learning, three essential parameters are indispensable: states, actions, and rewards. In specific scenarios, agents learn how to act most appropriately in a given situation by trying different actions to receive rewards or punishments. The problem is abstracted into models of states, actions, and rewards, transforming it into the question of “how to find the best states and actions”. In the Deep Q-network (DQN), rewards are crucial as they directly influence the learning process and the formation of strategies. DQNs evaluate the effects of different actions by receiving rewards from the environment. In short, rewards serve as key signals guiding agents in their decision-making during the training process.

According to the research context, in this study, we used the evaluation function of the mixing results (Equation (4)) to score the model’s predicted outcomes, thus defining the evaluation function for reinforcement learning. Different dimensional parameters correspond to various states of a mixer design, and evaluating the mixer design in that state completes the assessment of rewards. According to the theory of reinforcement learning algorithms, the trained agents can efficiently start from an initial state and quickly achieve the best design result state.

#### 2.4.3. Automated Design Process for Mixer Chips Based on Reinforcement Learning Algorithms

After defining the three pivotal concepts of state sets, action sets, and reward functions in the reinforcement learning training process, we utilized reinforcement learning algorithms to train the agents for configuring mixer structures. The design parameters of the mixer are treated as states within the reinforcement learning context. The mixer design 1 model features two design parameters (*a* and *b*), forming a two-parameter state set. The second model includes four parameters ($d_{1}$, $d_{2}$, $d_{3}$, $d_{4}$), resulting in a four-parameter state set.

The state set S and the current state st are mathematically expressed as follows:

(6) $$S_{t}=\left\{Param_{1},Param_{2},....,Param_{n}\right\}$$

In Equation (6), $Param_{1}$–$Param_{n}$ represent the design parameters of the mixer’s geometric structure, with *n* = 2 for the mixer design 1 model and *n* = 4 for the mixer design 2 model.

Let *A* denote the action set and at represent the current action. In the DQN framework, the agent determines the subsequent action at based on the current known state $s_{t}$ and ϵ−greedy.

The reward function is a critical parameter in the reinforcement learning algorithm. The agent adjusts its actions based on the current state, assigning a reward value rt. The objective of this section is optimization based on the target outlet concentration to find the optimal mixer design. The calculation formula for the mixing index, used as the main part of the reward function, is given by Equation (9). The difference in the mixing index between the DQN-optimized design and the COMSOL simulation model, along with the difference in the outlet concentration, collectively determines the magnitude of the reward value. A very small deviation results in a reward value of 1, while a larger discrepancy results in a penalty, setting the reward value at −1. The specific formula is as follows:

(7) $$\epsilon_{DQN}=1-\delta_{DQN}$$

(8) $$\epsilon_{COMSOL}=1-\delta_{COMCOL}$$

(9) $$\Delta \epsilon =|\epsilon_{\mathrm{DQN}}-\epsilon_{\mathrm{COMSOL}}|$$

(10) $$\Delta c=\frac{1}{N}\sum_{i=0}^{N}\left|c_{\mathrm{DQN},i}-c_{\mathrm{COMSOL},i}\right|$$

(11) $$rt=\left\{\begin{array}{cc} 1 & \mathrm{if}\;\Delta \epsilon <0.01\;\mathrm{and}\;\Delta c<0.01\\ 0.5 & \mathrm{if}\;0.01\leq \Delta \epsilon <0.05\;\mathrm{and}\;0.01\leq \Delta c<0.01\\ -1 & \mathrm{if}\;\Delta \epsilon >0.1\;\mathrm{and}\;\Delta c>0.05\\ 0 & \mathrm{otherwise} \end{array}\right.$$

**Figure 4:**
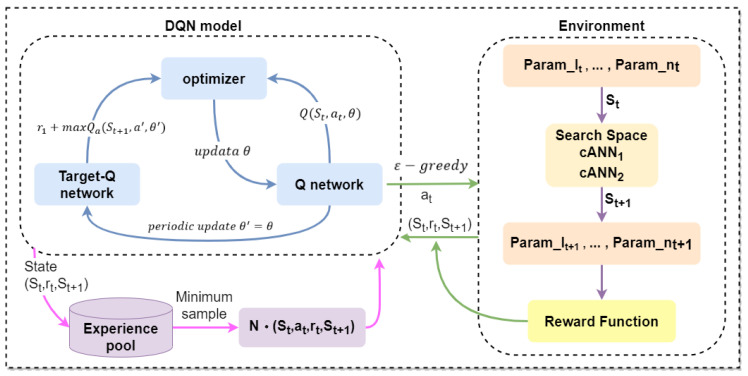
Implementation flowchart of DQN algorithm.

The optimization process of the DQN algorithm is illustrated in Figure 4, where two agents are trained for two different mixer design models to autonomously synthesize structural parameters. This training process includes two modules: the DQN agent training module and the environment interaction module. In the agent training module, the RMSProp algorithm is used to optimize the loss function, and two structurally identical ANNs are trained to predict the current Q-values and target-Q values, respectively. The network parameter configuration is shown in Table 2. The environment interaction module utilizes the low-overhead ANN model trained in Section 2.3 to obtain sample data.

Before the automated design algorithm commences, an initial design dimension is randomly selected as the starting state, and the current design parameters of the mixer are recorded in the current state set $s_{t}$. The Q-values for all actions under the state st are then output. Subsequently, the algorithm ϵ−greedy selects an action at based on the strategy, and inputs $a_{t}$ into the environment to obtain the new state $s_{t+1}$. The agent inputs the current structural design parameters into a pre-trained ANN model to predict the outlet concentration, and calculates the reward value based on the predicted concentration and the reward function. These data ($s_{t}$, $a_{t}$, $r_{t}$, $s_{t+1}$) are stored in the experience pool for subsequent training of the agent. Simultaneously, the DQN’s loss function and Q-value parameters are calculated and updated, as specified in Equation (12).

(12) $$L=\frac{1}{2}{\left[y_{t}-Q\left(s_{t},a_{t},\theta \right)\right]}^{2}$$

The Q-value parameters are periodically updated into the target-Q network, and the target values for the target-Q network are calculated as per Equation (13).

(13) $$y_{t}=r_{t}+\gamma^{*}\max Q\left(s_{t+1},a^{\prime },\theta^{\prime }\right)$$

The algorithm generates a vast amount of experience through continuous interaction with the environment, then randomly shuffles the data in the experience pool and selects small batches of data for training the Q-network. Ultimately, an optimal set of mixer design parameters is derived and output, completing one design cycle. The specific implementation process of the algorithm can be referred to in Figure 4.

## 3. Results and Discussion

Through experimental testing, the reinforcement learning-based automated design model proposed in this article successfully fulfilled the automated design requirements of the mixer designs. For two basic mixer designs, targeting the anticipated mixing efficiency, the agents trained using this algorithm can achieve superior final design outputs with fewer design iteration steps.

### 3.1. Simulation Library Building and Model Training

In the process of establishing a simulation dataset, the variable size parameters of the two types of mixer designs mentioned in Section 2.2 and the concentration values at 11 points of the corresponding outlets were transformed into matrices. Based on the variable parameter range specified in Table 1, the mixer design parameters were varied within a certain range. Ultimately, 10,000 design variants were explored for each mixer design, each of which was modeled using COMSOL 6.0, in the same simulation conditions, while we will keep the inlet fluid pressure constant throughout the simulation. The corresponding datasets and simulation models are stored in a local database for further use.

**Figure 5:**
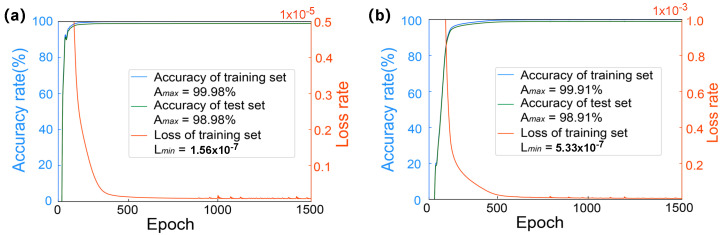
Two types of neural network training process diagrams: (**a**) cANN1 training process diagram; (**b**) cANN2 training process diagram.

After collecting the dataset and performing data preprocessing, we utilized the cleaned data to train high-precision, low-overhead models for two structural bodies. The training processes of these two artificial neural networks are depicted in Figure 5. Of the 10,000 datasets, 70% were used as the training set, with the remaining 30% serving as the test set. Figure 5a illustrates the training process of the neural network cANN1 for mixer design 1, which, after 1500 iterations of training, achieved a training accuracy of 99.98% and a test accuracy of 98.98%, with the minimum loss rate in the training set being 1.56 × 10^−7^. Figure 5b shows the training process of the neural network cANN2 for mixer design 2, which, after 1500 iterations, reached a training accuracy of 99.91% and a test accuracy of 98.91%, with the minimum loss rate in the training set being 5.33 × 10^−7^.

**Figure 6:**
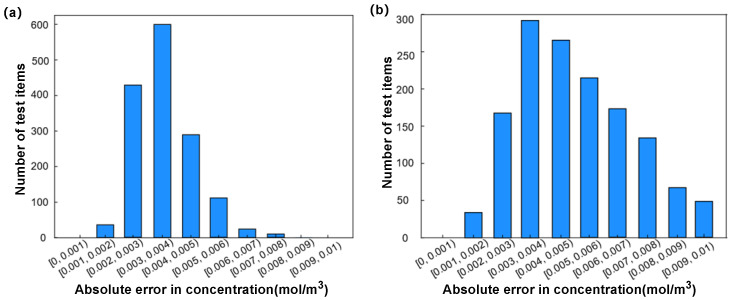
The absolute error distribution diagram of cANN test set: (**a**) represents cANN1; (**b**) represents cANN2.

To validate the training efficacy of these two artificial neural networks, we utilized finite-element simulation software to re-simulate 1500 models for each mixer design as a test set, employing cANN1 to predict the outlet concentration of mixer design 1. Figure 6a displays the absolute errors in outlet concentration for the test set of mixer design 1 (the difference between the target values and the concentrations predicted by cANN), where 11.2%, 19.4%, and 17.7% of the concentration data points had absolute errors less than 0.003 mol/m^3^, 0.004 mol/m^3^, and 0.005 mol/m^3^, respectively. When the absolute error threshold was increased to 0.009 mol/m^3^, the percentage rose to 85.5%.

For mixer design 2, the absolute errors in the outlet concentration of the test set are shown in Figure 6b, where 28.6%, 40%, and 19.3% of the concentration data points had absolute errors less than 0.003 mol/m^3^, 0.004 mol/m^3^, and 0.005 mol/m^3^, respectively. All 1500 model data points had absolute errors within 0.009 mol/m^3^.

### 3.2. Design Results of Two Mixers’ Size Parameters Based on Reinforcement Learning Algorithm

Utilizing the DQN framework from the automated mixer design algorithm proposed in this paper, we conducted 50 sets of optimization designs for the two classic mixer structures mentioned in Section 2.1 (data shown in Table 3 and Table 4). The training process of the agent and the post-simulation verification of the mixer designs were conducted on a computer running Windows 10, equipped with an Intel Core i7-10700 CPU and 64 GB of RAM.

#### 3.2.1. Automated Design Results of Structure 1

**Figure 7:**
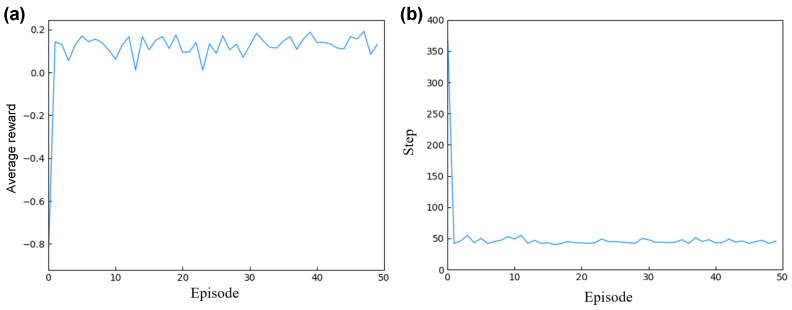
Mixer design 1 intelligent agent training and design details: (**a**) describes the training process diagram of the mixer design 1 DQN; (**b**) describes the optimization steps for each episode in the mixer design 1 DQN.

As illustrated in Figure 7, the parameter design for mixer design 1’s structure is detailed, showcasing the training and design specifics of the intelligent agent. Figure 7a depicts the convergence process of the DQN optimization algorithm, where the *x*-axis represents the number of training iterations and the *y*-axis indicates the average reward per iteration. Initially, the agent’s average reward was minimal and negative. However, through continuous interaction between the agent and the environment, the calculation of the reward function, and adjustment of the action decisions, the score converged after 50 iterations, culminating in a set of optimized parameters.

During the training of the agent, each episode’s optimization design steps were recorded, as shown in Figure 7b. The *x*-axis represents the number of design iterations per episode, and the *y*-axis corresponds to the steps taken to complete the design. Initially, optimizing a set of design parameters required several hundred iterations to produce the best design outcome. However, as the agent effectively learned from the data in the experience pool, it was able to optimize a set of design parameters in just 40 iterations post-training, significantly speeding up the design process.

**Figure 8:**
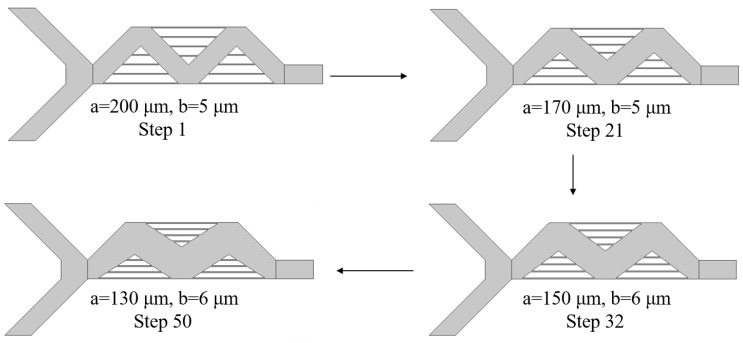
Process result for adjusting the structure of mixer design 1.

From the 50 sets of optimization designs, several were randomly selected to exemplify the optimization process of the DQN intelligent agent for the structural size parameters of mixer design 1. Figure 8 randomly selects four steps from the total 50-step iterative process, demonstrating how the agent adjusted the structural design parameters. The final design was optimized with parameters a = 130 µm and b = 6 µm. Initially, the values of a and b were set at 200 µm and 5 µm, respectively. Throughout the interaction with the environment, the agent continuously learned and optimized its strategy, adjusting the structural design actions. By the 21st step, the parameters were adjusted to a = 170 µm and b = 5 µm. Using the cANN1 to predict the new mixer design structure performance and calculating the reward value based on the reward function, the agent proceeded to the next iteration, ultimately achieving the optimal design of a = 130 µm and b = 6 µm after 50 iterations.

**Figure 9:**
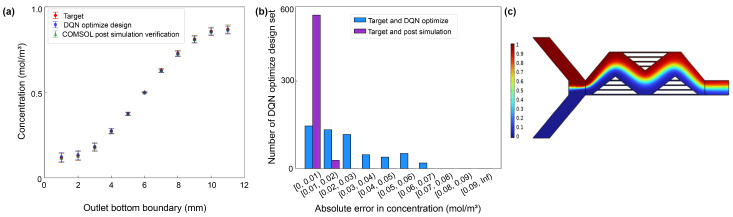
Comparison of outlet concentration values for mixer design 1 based on DQN optimization design and COMSOL post-simulation verification: (**a**) A comparative graph of the concentration values between the two methods. (**b**) Distribution graphs of absolute errors in concentration between the target values and both the DQN-optimized design and the COMSOL post-simulation verification. (**c**) Mixed results chart.

Upon the completion of the design, the 50 sets of optimized design parameters underwent post-simulation verification in COMSOL. A comprehensive statistical analysis of the DQN optimization design’s performance was then conducted. This analysis compared the outlet concentration values of the mixer, the target concentration values, and the post-simulation verified outlet concentration values from COMSOL, as illustrated in Figure 9.

In Figure 9a, the *x*-axis represents 11 points at the mixer’s outlet, while the *y*-axis depicts the concentration, spanning from 0 to 1. The red diamond labels stand for the mean of the target concentration values, with the variance at that location indicated by the fluctuation above and below the mean. The blue circle labels represent the mean of the DQN optimized design’s outlet concentration, along with its variance. Similarly, the green triangle labels indicate the mean of the COMSOL post-simulation verified outlet concentration, also with its variance. The overlapping of these labels suggested that the variance-calculated outlet concentration values of the mixer are very close, indicative of minimal error.

Figure 9b depicts the absolute errors between the target concentration values and the DQN-optimized design concentration values, as well as between the target concentration values and the COMSOL post-simulation verified concentration values. The *x*-axis showcases the error range, and the *y*-axis represents the number of occurrences in each interval. The distribution of absolute errors between the target concentration values and the post-simulation verified values shows that more than 94.9% of the concentration points had an absolute error less than 0.01 mol/m^3^, with 5.1% having an error less than 0.02 mol/m^3^. On the other hand, the distribution of the absolute errors between the target concentration values and the DQN-optimized design indicated that more than 26.4%, 24%, and 21.1% of the concentration points had an absolute error less than 0.01 mol/m^3^, 0.02 mol/m^3^, and 0.03 mol/m^3^, respectively.

The post-simulation verification visually demonstrates that the automated design process for the mixer design 1 structure performed excellently. The final output of the design meets the design requirements, and the overall design time was significantly reduced compared to a manual design. This indicates that the proposed algorithm’s reinforcement learning approach effectively meets the needs for ensuring design effectiveness and enhancing design efficiency in the automated design of this structure’s dimensions.

#### 3.2.2. Automated Design Results of Structure 2

**Figure 10:**
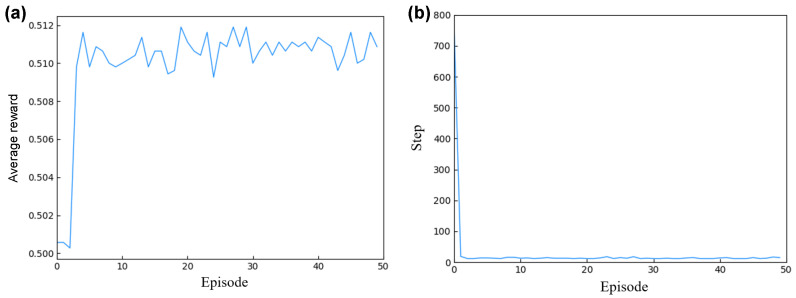
Mixer design 2 intelligent agent training and design details. (**a**) describes the training process diagram of mixer design 2 based on the DQN; (**b**) describes the optimization step iteration diagram of mixer design 2 based on the DQN.

For mixer design 2, the training process is illustrated in Figure 10. The *x*-axis represents the number of iterations of training, while the *y*-axis denotes the average reward value per iteration. Figure 10a describes the convergence process of the DQN optimization algorithm. Initially, the agent’s average reward values were low. Through interaction with the simulation environment and utilizing cANN2 to predict the mixer design outlet concentration values based on the current structural design parameters, as well as calculating corresponding the reward values, the agent adjusted its actions accordingly. After 50 iterations, the agent quickly converged, ultimately yielding a set of optimized parameters.

During the training of the agent, the number of steps taken to optimize a set of design parameters per episode was recorded, as shown in Figure 10b. The *x*-axis is the number of episodes, and the *y*-axis is the corresponding number of steps. Initially, optimizing a set of design parameters required over seven hundred iterations per episode. However, as the agent effectively learned from the data in the experience pool, it required only 12 iterations to rapidly complete the optimization of a set of design parameters after training.

**Figure 11:**
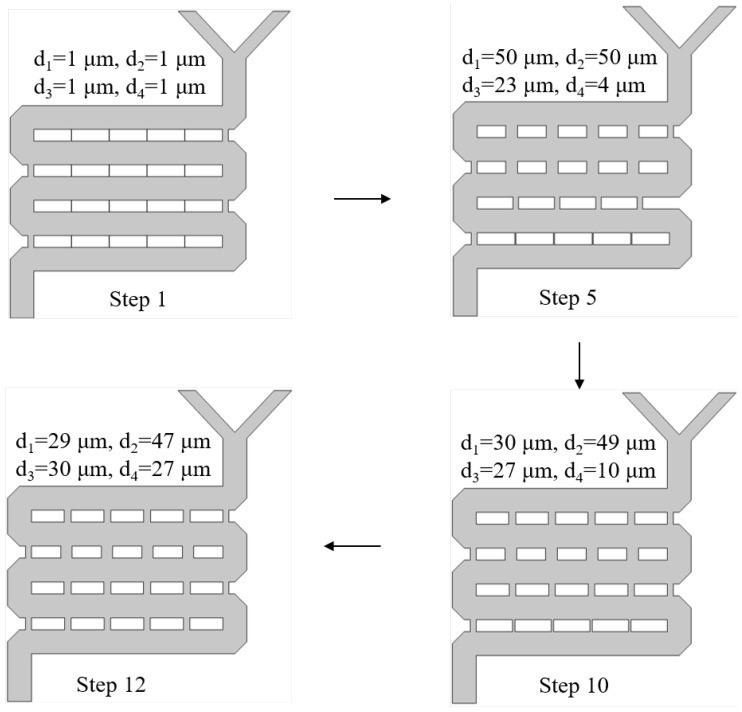
Mixer design 2 structure adjustment process.

From a selection of twelve optimized designs, several were chosen at random to exemplify the process by which the DQN agent optimizes the geometric structure of mixer design 2 using the reinforcement learning algorithms, based on the target structure’s outlet concentration values. The adjustable parameters for this mixer design are the widths of four microchannels, designated as $d_{1}$, $d_{2}$, $d_{3}$, and $d_{4}$, with all other parameters remaining constant. Figure 11 illustrates the process of the agent adjusting the structural design parameters. Four steps were randomly selected from a twelve-step design process. The initial design parameters were set at $d_{1}$, $d_{2}$ = 1 µm, $d_{3}$ = 1 µm, and $d_{4}$ = 1 µm. Throughout the interaction with the environment, the agent continuously learned and optimized its strategy, adjusting its actions based on the structural design. Utilizing cANN2, the agent predicted the performance of new mixer design structures and calculated reward values based on the reward function before proceeding to the next iteration. Ultimately, after twelve iterations of design, the agent achieved an optimal design with parameters $d_{1}$ = 29 µm, $d_{2}$ = 47 µm, $d_{3}$ = 30 µm, and $d_{4}$ = 27 µm.

**Figure 12:**
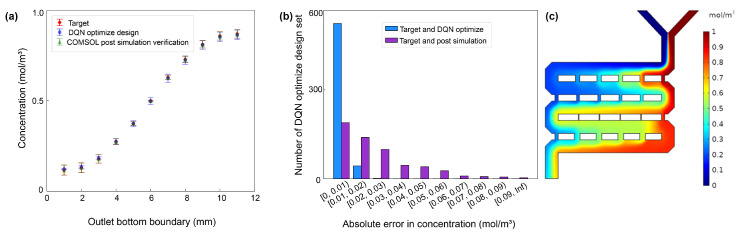
Comparison of mixer design 2 based on DQN optimization design, COMSOL post-simulation verification, and target outlet concentration values: (**a**) Concentration comparison chart for the three methods. (**b**) Distribution chart of absolute errors in concentration between the target values and both the DQN-optimized design and COMSOL post-simulation verification. (**c**) Mixed results chart.

Upon the completion of the design phase, the optimized parameters for the final set of twelve mixer designs were utilized in COMSOL for simulation calculations. Figure 12 provides a statistical analysis of the performance of the DQN-optimized designs, comparing the optimized concentration values of mixer design 2, the target concentration values, and the concentration values verified by COMSOL post-simulation. In Figure 12a, the *x*-axis represents 11 points at the mixer design outlet, while the *y*-axis represents the concentration, ranging from 0 to 1. The red diamond labels denote the mean of the target concentration values, with variance representing the fluctuation at that position; the blue circle labels indicate the mean of the DQN-optimized outlet concentration, with variance as the fluctuation; the green triangle labels represent the mean of the post-simulation verified outlet concentration, with variance as the fluctuation. The substantial overlap among the three labels signifies a high level of consistency between the DQN-optimized design, the post-simulation verified mixer design, and the target design in terms of outlet concentration.

Figure 12b displays the absolute errors between the target concentration values and the DQN-optimized concentration values, as well as between the target concentration values and the post-simulation verified concentration values. The *x*-axis represents the range of errors, while the *y*-axis illustrates the number of points in each interval. In the distribution of absolute errors between the target concentration values and the post-simulation verified concentration, more than 33.3%, 24.5%, and 17.5% of the concentration points exhibit absolute errors of less than 0.01 mol/m^3^, 0.02 mol/m^3^, and 0.03 mol/m^3^, respectively. In the distribution of absolute errors between the target concentration values and the DQN-optimized concentration, over 92.2%, 7.6%, and 0.1% of the concentration points have absolute errors less than 0.01 mol/m^3^, 0.02 mol/m^3^, and 0.03 mol/m^3^, respectively.

From the post-simulation verification, it can be concluded that the automated design process for the second type of mixer structure performed excellently. The final output of the design meets the design requirements, and the overall design process significantly improved in terms of time consumption. This indicates that the reinforcement learning algorithm proposed in this paper satisfies the needs to ensure design effectiveness and enhance design efficiency.

## 4. Potential Limitations

There are still shortcomings and limitations that require continuous exploration and improvement:

(1) Agents obtained through reinforcement learning can only optimize designs for trained structures and cannot use a single agent to achieve universal design across multiple structures. This means that designing multiple structures would require training multiple agents, significantly increasing the technical investment in the early stages of design.
(2) Currently, the design capabilities of microfluidic mixer agents are based on training with predictive data from high-accuracy, low-overhead ANN models. This means that the predictive accuracy of the ANN models directly affects the design capabilities of the agents. Moreover, beyond the predictive capabilities of the ANN models, the design capabilities of the agents are also significantly reduced.
(3) In this experiment, the fluid simulation experiments were executed under the set fixed boundary conditions. Although this approach cuts down the influence of the Reynolds number in scoring the design results, it also limits the applicability of our validation algorithm to different flow conditions. As a result, we can only conclude that the proposed algorithm is valid in the tested flow rate range. This limitation undoubtedly poses a constraint on our ability to fully evaluate the performance of the algorithm. In subsequent studies, we will continue to explore the performance of the algorithm under different boundary conditions to verify its generality.

## 5. Conclusions

This study explores an automated method for synthesizing micromixer design parameters using a reinforcement learning algorithm based on the DQN framework. Tests were conducted on the structural designs of two classic micromixers. During the algorithm testing, reward functions were designed based on the formula for mixing efficiency of micromixers, considering the variable structural parameters and corresponding outlet concentration values. Combined with a pre-trained low-overhead artificial neural network model, two agents were trained to automatically optimize the design of the two types of micromixers. Using a dataset of 50 groups, the two agents were trained to automatically optimize the designs of these classic micromixers. The experiments demonstrated that a single call to the agent for automated optimization design took 0.129 s for the first type of micromixer and 0.169 s for the second type, significantly faster than manual design simulation times.

Unlike other optimization-algorithm-driven automated design processes, the intelligent design using reinforcement learning does not easily fall into the trap of local optima. Agents trained using reinforcement learning technology are capable of analyzing the current state in a more thoughtful manner and are oriented towards optimal goals, seeking the best solution under current design requirements. Such a design process is forward-looking in the direction of the automated design of micromixer chips.

## Figures and Tables

**Table 1:** Range of design parameters for two micromixers.

Mixers	Design Parameters	Range/µm
Mixer design 1	*a*	100 < *a* < 200
	*b*	5 < *b* < 15
Mixer design 2	$d_{1}$	1 < $d_{1}$ < 50
	$d_{2}$	1 < $d_{2}$ < 50
	$d_{3}$	1 < $d_{3}$ < 50
	$d_{4}$	1 < $d_{4}$ < 50

**Table 2:** Configuration parameters for Q-network and target-Q network of two intelligent agents.

	Input Layer	Activation Function	Neuron Node Count	Output Layer
IntelligentAgent1	2	ReLU	11	11
IntelligentAgent2	4	ReLU	11	11

**Table 3:** The concentration mean and variance of 11 points of 50 target concentration values for mixer design 1.

	$c_{0}$	$c_{1}$	$c_{2}$	$c_{3}$	$c_{4}$	$c_{5}$	$c_{6}$	$c_{7}$	$c_{8}$	$c_{9}$	$c_{10}$
Mean	0.117	0.129	0.179	0.273	0.374	0.500	0.629	0.729	0.812	0.858	0.869
Variance	0.027	0.027	0.023	0.016	0.010	0.003	0.011	0.017	0.020	0.023	0.023

**Table 4:** The concentration mean and variance of 11 points of 50 target concentration values for mixer design 2.

	$c_{0}$	$c_{1}$	$c_{2}$	$c_{3}$	$c_{4}$	$c_{5}$	$c_{6}$	$c_{7}$	$c_{8}$	$c_{9}$	$c_{10}$
Mean	0.333	0.337	0.357	0.399	0.455	0.510	0.559	0.598	0.624	0.635	0.637
Variance	0.024	0.023	0.020	0.016	0.012	0.006	0.008	0.016	0.023	0.026	0.026

## Data Availability

The original contributions presented in the study are included in the article, further inquiries can be directed to the corresponding authors.
